# Testing frameworks for personalizing bipolar disorder

**DOI:** 10.1038/s41398-017-0084-4

**Published:** 2018-02-02

**Authors:** Amy L. Cochran, André Schultz, Melvin G. McInnis, Daniel B. Forger

**Affiliations:** 1 Department of Biostatistics and Medical Informatics, University of Wisconsin, Madison, WI 53705 USA; 2 Department of Bioengineering, Rice University, Houston, TX 77030 USA; 3 Department of Psychiatry, University of Michigan, Ann Arbor, MI 48105 USA; 4 Department of Mathematics, University of Michigan, Ann Arbor, MI 48105 USA; 5 Department of Computational Medicine and Bioinformatics, University of Michigan, Ann Arbor, MI 48105 USA

## Abstract

The hallmark of bipolar disorder is a clinical course of recurrent manic and depressive symptoms of varying severity and duration. Mathematical modeling of bipolar disorder holds the promise of an ability to personalize diagnoses, to predict future mood episodes, to directly compare diverse datasets, and to link basic mechanisms to behavioral data. Several modeling frameworks have been proposed for bipolar disorder, which represent competing hypothesis about the basic framework of the disorder. Here, we test these hypotheses with self-report assessments of mania and depression symptoms from 178 bipolar patients followed prospectively for 4 or more years. Statistical analysis of the data did not support the hypotheses that mood arises from a rhythmic process or multiple stable states (e.g., mania or depression) or that manic and depressive symptoms are highly anti-correlated. Alternatively, it is shown that bipolar disorder could arise from an inability for mood to quickly return to normal when perturbed. This latter concept is embodied by an affective instability model that can be personalized to the clinical course of any individual with chronic disorders that have an affective component.

## Introduction

Bipolar disorder (BP) is a chronic illness of recurrent episodes of mania and depression, affecting 2.4% of the adult population^1,2^. This disorder is classified according to diagnostic criteria in the Diagnostic and Statistical Manual 5th Edition (DSM-5)^3^, which are largely based on expert consensus through empirical clinical observations. These criteria lose information on sub-syndromal symptoms and the dynamic nature of the illness beyond simple observations of episodic pattern. There is much interest in further quantifying BP through mathematical modeling.

Several models have recently been proposed for BP. They are built upon the following hypotheses:

Bipolar is intrinsically rhythmic: A periodic assumption postulates mood is driven by an internal timekeeping mechanism. As a result, mood cycles through mania and depressive episodes rhythmically or periodically. Periodic models are readily available for BP^4–9^.

Bipolar is multistable: A multistable assumption argues mood in BP tends to distinct mood states (e.g., mania and depression) which sustain mood at severe levels. Multistability is captured mathematically with stable points or attractors in dynamical systems^7,8,10–12^.

Bipolar is one-dimensional: A one-dimensional assumption supposes mood is a spectrum with mania and depression on opposite ends (i.e. the two “poles” in bi-polar). Manic or depressive symptoms arise only in the absence of the other. Many models of mood in BP use a one-dimensional assumption^4,5,9,11,13^.

Testing these hypotheses could have major implications for the study of BP. The multistable hypothesis suggests that environmental or internal perturbations, “stress” or “noise,” are what triggers a mood episode. Thus, if these could be minimized, mood would stay in its current state indefinitely. Likewise, when the next episode is, or its duration, is fundamentally unpredictable. The rhythmic hypothesis suggests the opposite: that mood transitions could occur independently of these perturbations and that mood episodes are fundamentally predictable and have a characteristic duration. A third hypothesis is that mood episodes are triggered by these perturbations, but that these mood states are not “sustaining” or that there is an effective maximal time by which the mood episode would ultimately end, perhaps because of an exponential return to a baseline mood.

The one-dimensional hypothesis suggests that any increase in manic symptoms means a decrease in depressive symptoms. Even if a model allows for two separate variables, it could “attract” to a state where this relationship between manic and depressive symptoms is true. An alternative is to assume manic and depressive scores are independent. A middle ground exists where mania and depression are “correlated” (positively or negatively).

In this study, we aimed to (i) formally test the validity of these hypotheses at the patient-level, and subsequently (ii) establish a mathematical framework for clinical course in BP. In what follows, patient-level statistical tests are combined across patients to examine collective evidence of each hypothesis. In the process, we reveal evidence of a new model that describes mood course in BP as arising from extreme instability in manic and depressive symptoms. We then show how this model can be personalized to the mood course of any individual with a chronic condition wherein mood and affective symptoms are present (not just bipolar patients), thereby providing a quantitative phenotype to study biological mechanisms of disorders that manifest, at least in part, with affective symptoms.

## Materials and methods

### Data

The primary dataset, the *bimonthly dataset*, was collected from 178 BP individuals followed prospectively for at least 4 years in the Prechter Longitudinal Study of Bipolar Disorder at the University of Michigan^14^. An Altman Self-Reported Mania scale (ASRM)^15^ and the Patient Health Questionnaire for Depression (PHQ9)^16^ were completed at 2 month intervals. On average across the individuals, 3.5% of PHQ9 scores were missing and 0.55% of ASRM were missing. Of the 178 individuals, 138 were BPI, 12 were BP not otherwise specified, and 28 were BPII; 134 were female; 153 individuals were white, 6 black or African-American, 2 Asian, 9 more than one race, and 8 patients of unknown race; 166 individuals were not-Hispanic, 5 Hispanic, and 7 of unknown ethnicity. Individuals were on average 42.3 ± 12.2 (±standard deviation [SD]) years of age at the initial interview for the Prechter study. The UM IRB approved recruitment, assessment, and research procedures (HUM606).

A second dataset, the *weekly dataset*, was included for two statistical tests that depend on how often mood was sampled. This dataset was collected on BP individuals (*N* = 15) from the Prechter Study with at least 24 surveys scores on the Young Mania Ratings Scale (YMRS)^17^ and the Structured Interview Guide from the Hamilton Depression Rating Scale (SIGHD)^18^, administered weekly by trained interviewers. On average across the individuals, 9.2% of SIGHD scores were missing and 9.7% of YMRS score were missing. Of these patients, 9 were BPI and 6 were BPII; 12 were female; 9 were white, 1 Asian, 4 black or African-American, and 1 of unknown ethnicity; 15 were not-Hispanic. They were on average 39.9 ± 9.4 (±SD) years of age at the initial interview. Ten patients had both bimonthly and weekly data.

### Statistical approach

Because the hypotheses of interest are about BP at *the patient-level*, they should be tested with patient-level data. Since patient-level data is limited and highly variable, patient-level tests may not have sufficient statistical power to reject hypotheses. Testing hypotheses on aggregated data across patients, however, can lead to wrong conclusions about patient-level trends. To overcome this limitations, we perform statistical tests on patient-level data, but then aggregate statistics and/or test results across patients. Significance was considered an alpha level of 0.05, and the analysis was performed in Matlab (Mathworks; Natick, MA) unless otherwise specified.

### Patient-level statistics

#### Testing a one-dimensional hypothesis

Kendall rank correlation was measured between concurrent depressive and manic symptoms (bimonthly dataset). Rank correlation measures the degree to which an increase in depressive symptoms is accompanied by an increase in mania and similarly, an increase in manic symptoms is accompanied by an increase in depression. Because a one-dimensional hypothesis ignores mixed states, we also compared risk for mania (ASRM score ≥ 6) while depressed (PHQ9 score ≥ 10) versus not depressed (PHQ9 score < 10) (bimonthly dataset). We ignored ill-defined rank correlations (an individual’s survey scores are all identical) and ill-defined risk values (an individual was either never depressed or never not depressed).

#### Testing a rhythmic hypothesis

An individual’s scores were transformed to the frequency domain using nonparametric spectral estimation based on Thomson’s multitaper approach^19^. We then looked for significant oscillation frequencies using Thomson’s harmonic *F* test, from which we could recover *P*-values for each frequency (Null hypothesis i: *An individual’s manic or depressive scores do not oscillate at a specified frequency;* bimonthly and weekly datasets). Thirty frequencies were tested, equally spaced between 1/24 and 1/2 of the sampling frequency, i.e., between 1/4 and 3 cycles per year for the bimonthly dataset and 1/24 and 1/2 cycles per week for the weekly dataset. Spectral estimation was performed in R (R Foundation for Statistical Software, Vienna, Austria) using the multitaper package with seven Slepian tapers, a time bandwidth of four, adaptive estimation, and removing the estimated mean of the time series using Slepian tapers. This analysis required contiguous data, so the R function na.approx (found in the zoo package) filled in missing data via an interpolation method.

#### Testing a multistable assumption

To test for multiple states, we looked for survey scores with probability density functions that had multiple modes, i.e., particular mood scores that are relatively more common than nearby scores^20^. A Hartigan’s dip test was used to measure deviation from unimodality^21^ (null hypothesis ii: *an individual’s manic or depressive scores has a unimodal distribution;* bimonthly dataset). Since a Hartigan’s dip test requires continuous data and survey scores are discrete, we added a uniform random variable to each score and applied the dip test to these modified scores.

#### Testing our affective instability model

We tested the validity of our model by evaluating whether it fits survey scores *significantly worse* than data sampled from the model, i.e. whether we can distinguish between actual data and data simulated from the model. This approach does not favor model complexity: even if a complex model fits the data better, it is not guaranteed that the model fits the data better than simulated data. This testing relied on two types of goodness-of-fit tests, following a method to validate models for financial data described by Ait-Sahalia et al.^22^ and Fan^23^ (see Supplementary Appendix for details). The first goodness-of-fit test evaluated how well certain probability density functions fit survey scores. (Null hypothesis iii: *an individual’s manic or depressive scores are drawn from a specified probability density function;* monthly dataset). The second goodness-of-fit test evaluated how well certain transition distributions fit sequences of mood scores (Null hypothesis iv: *an individual’s sequence of manic or depressive scores are drawn from a specified transition distribution;* weekly dataset). For the latter test, the weekly dataset was used, since bimonthly scores were not sampled frequently enough to accurately estimate parameters. We measured goodness-of-fit to the density function and transition distribution defined by our model. To show these tests have enough statistical power to reject models, we also tested two common stochastic differential equation models: an Ornstein-Uhlenbeck process and a Cox-Ingersoll Ross process^24^. Since our model is unistable under no noise, these tests also serve to further test multistability.

### Population-level statistics

Statistical tests were aggregated to examine collective evidence of each patient-level null hypothesis. For tests with *P*-values, we followed Loughin^25^, calculating a mean *P*-value across patients for each test and testing for significance by comparing it to the mean *P*-value of the same number of independent uniform random variables (Null hypothesis I: *the null hypothesis of a particular test holds across patients, and tests are independent between patients*; bimonthly and weekly datasets). Under this approach, a hypothesis would not be rejected with one *P*-value of 0.2, but would be rejected with one-hundred *P*-values of 0.2.

Since multiple frequencies are tested (leading to a higher chance for Type I errors) and mood could oscillate at different frequencies between patients, we also calculated the minimum of the *P*-values across the 30 frequencies for each individual. These minima were averaged and compared to a similar statistic assuming *P*-values across individuals and frequencies were independent uniform random variables. (Null hypothesis II: *the null hypothesis of a Thomson’s F tests holds across patients and frequencies, and tests are independent between frequencies and patients;* bimonthly and weekly datasets).

To clearly associate a population-level statistical test to a reported *P*-value, we use scalars $\hat P_i$, $\hat P_{ii}$, $\hat P_{iii},\,\hat P_{iv}$ to denote *P*-values recovered under the population-level null hypothesis I, which in turn are associated with null hypotheses i, ii, iii, or iv. In addition, $\tilde P_i$ denotes the *P*-value recovered under *population-level* null hypothesis II.

### Parametric study

To analyze our affective instability model, we estimated mean duration of mood episodes and percent time in a mood state for certain parameters. To remove the dependence of these estimates on initial mood values and random noise, we followed a common strategy in stochastic simulation by simulating the affective instability model for a sufficiently long period of time with a suitable warm-up or burn-in period^26^. With mood episodes lasting on a scale of weeks to months, we chose to simulate the model for 1100 simulated life-years with initial manic/depressive mood values of 0.1 and warm-up period of 100 years, storing daily samples of mood for only the last 1000 simulated life-years. We are in no way assuming that individuals live for 1000 years, but use this value simply to remove any dependence on initial conditions and random noise.

Because mood is continuous in the affective instability model, threshold values of mood were chosen to separate mood into states. Because mood is also dimensionless, any threshold value can be used and gains an interpretation when compared to other parameters and when mood is scaled to match a survey. So, we chose a threshold value of 3 to define mood states:

- Euthymia: manic and depressive variables less than 3;
- Mania: manic variable greater than 3 and a depressive variable less than 3;
- Depression: manic variable less than 3 and a depressive variable greater than 3; and
- Mixed State: manic and depressive variables greater than 3.

Manic, depressive, and mixed episodes were defined as periods in the corresponding mood state lasting at least a week to agree with current DSM guidelines; euthymic episodes were periods in-between mood episodes.

## Results

### Testing conventional hypotheses

#### One-dimensional

**Fig. 1:**
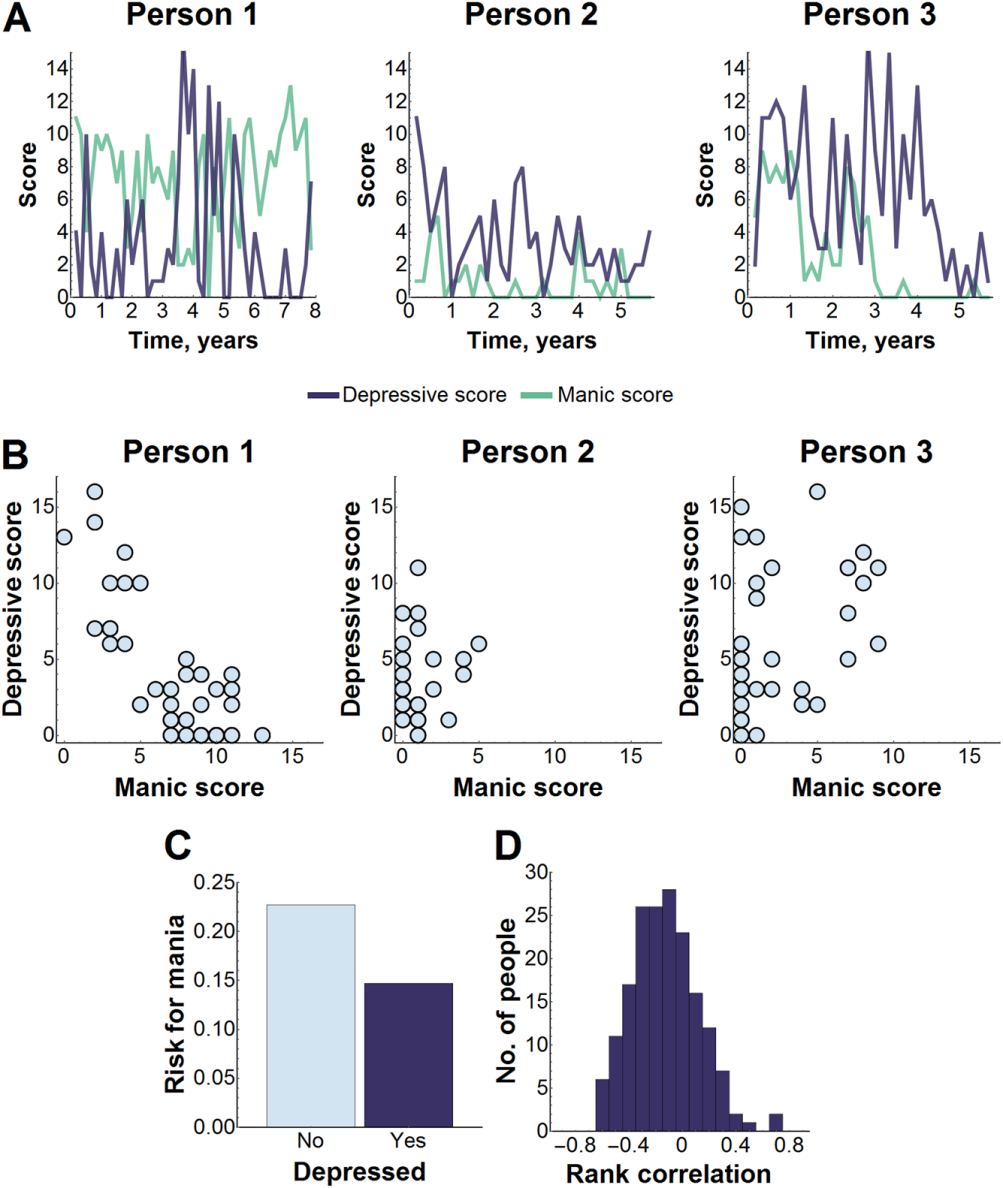
Testing a one-dimensional hypothesis. **a** Depressive and manic surveys were collected bimonthly in individuals with BP. Mood is highly variable and only a limited amount of data is available on each individual, so patient-level hypotheses cannot be formally tested from visual inspection of mood course alone. **b** Manic scores were plotted against concurrent depression scores. If mood is one-dimensional, concurrent manic and depressive symptoms for *all* patients would have been plotted near a one-dimensional curve and depressive symptoms would never arise with manic symptoms, indicated by negative correlation between the two symptom types. **c** Average risk for mania (ASRM score ≥ 6) across individuals was as high as 15% when depressed (PHQ9 score ≥ 10), close to the 23% risk for mania when not depressed. **d** Rank correlation was measured in 178 patients to evaluate the degree to which manic symptoms were negatively correlated with depressive symptoms. Persons 1–3 were chosen to illustrate the range of rank correlations from negative to near-zero to positive

A one-dimensional hypothesis would mean that individuals are never both manic and depressed (Fig. 1a, b). However on average, individuals in our dataset have a 15% risk for mania (ASRM score ≥ 6) while depressed (PHQ9 score ≥ 10) (Fig. 1c). Interestingly, an individual has only a 23% risk for mania while not depressed. The significant risk for mania while depressed goes against the one-dimensional hypothesis.

To test this further, because a one-dimensional hypothesis requires that high manic scores would occur only with low depressive scores and high depressive scores occur only with low manic scores, a parametric plot of manic and depressive survey scores would appear near a one-dimensional curve in each individual (Fig. 1b). We measured Kendall’s rank correlation between depressive and manic scores for each individual (Fig. 1d) to tell if mania and depression are negatively correlated (i.e. high manic scores are found with low depressive scores and vice versa), or positively correlated, which would lead to mixed episodes. The rank correlation was on average −0.13 across individuals and ranged from −0.60 to 0.75. While some individuals (e.g., Person 1 in Fig. 1b) showed a negative correlation close to 1, this was not typical. Positive correlation for some individuals further contradicts the idea that mania and depression are mutually-exclusive and the one-dimensional hypothesis.

#### Rhythmic

**Fig. 2:**
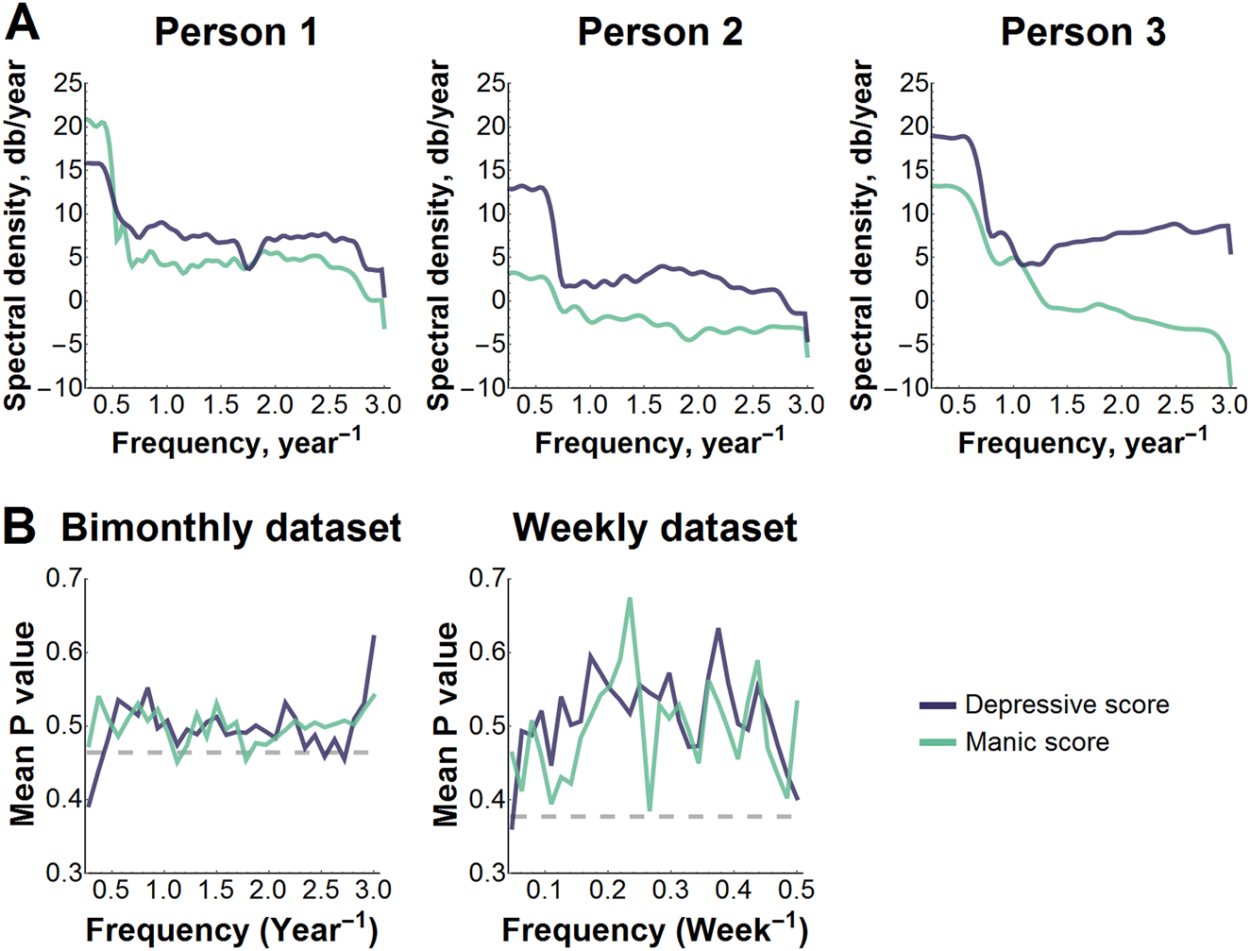
Testing a rhythmic hypothesis. **a** Mood scores were transformed into the frequency domain using spectral density estimation. A rhythmic mood course would result in a significant peak in the spectral density. **b** Frequencies were tested for significance and then the resulting *P*-values were averaged across patients to evaluate the collective evidence that depressive and manic scores in the weekly (*N* = 178) and bimonthly (*N* = 15) datasets oscillate at a particular frequency. Significance is a mean *P*-value that dips below the dashed lines, which mark 5th quantiles for the mean of *N* uniform random variables between 0 and 1 with *N* = 178 for the bimonthly dataset and *N* = 15 for the weekly dataset

Under a rhythmic assumption, an individual’s mood scores transformed to a frequency domain peak around a particular frequency (Fig. 2a). We tested frequencies for significance and combined these tests across individuals into mean *P*-values, leading to equivocal evidence of rhythmicity (Fig. 2b). Near the fundamental frequencies (the reciprocal of the observation period), mean *P*-values were low enough across patients to be significant for both datasets. However, the fundamental frequency should be significant, since spectral estimation requires repeating the data every observation period. For the weekly dataset, mean *P*-values were not low enough to suggest mood oscillates at any other frequencies.

For the bimonthly data, mean *P*-values were low enough across patients to suggest manic symptoms oscillate at 1.13 and/or 1.78 cycles per year ($\hat P_i$ = 0.028 and 0.020), but not depressive symptoms ($\hat P_i$ = 0.47 and 0.63), and to suggest depressive symptoms oscillate at 2.53 and/or 2.72 cycles per year ($\hat P_i$ = 0.012 and 0.017), but not manic symptoms ($\hat P_i$ = 0.12 and 0.34). Manic and depressive symptoms might thus oscillate at different frequencies that may not correspond to any biological (e.g. 4 week periods) or seasonal oscillation (e.g. one or six month periods). However, we tested 30 frequencies for significance, which increases the possibility of falsely concluding a frequency is significant when it is not. When we correct for testing multiple frequencies and allow mood to oscillate at different frequencies between individuals, we find insufficient evidence to suggest that manic or depressive symptoms oscillate in the weekly or monthly datasets ($\tilde P_i$ > 0.19). In sum, it is unclear if manic and depressive symptoms oscillate from our datasets.

#### Multistable

**Fig. 3:**
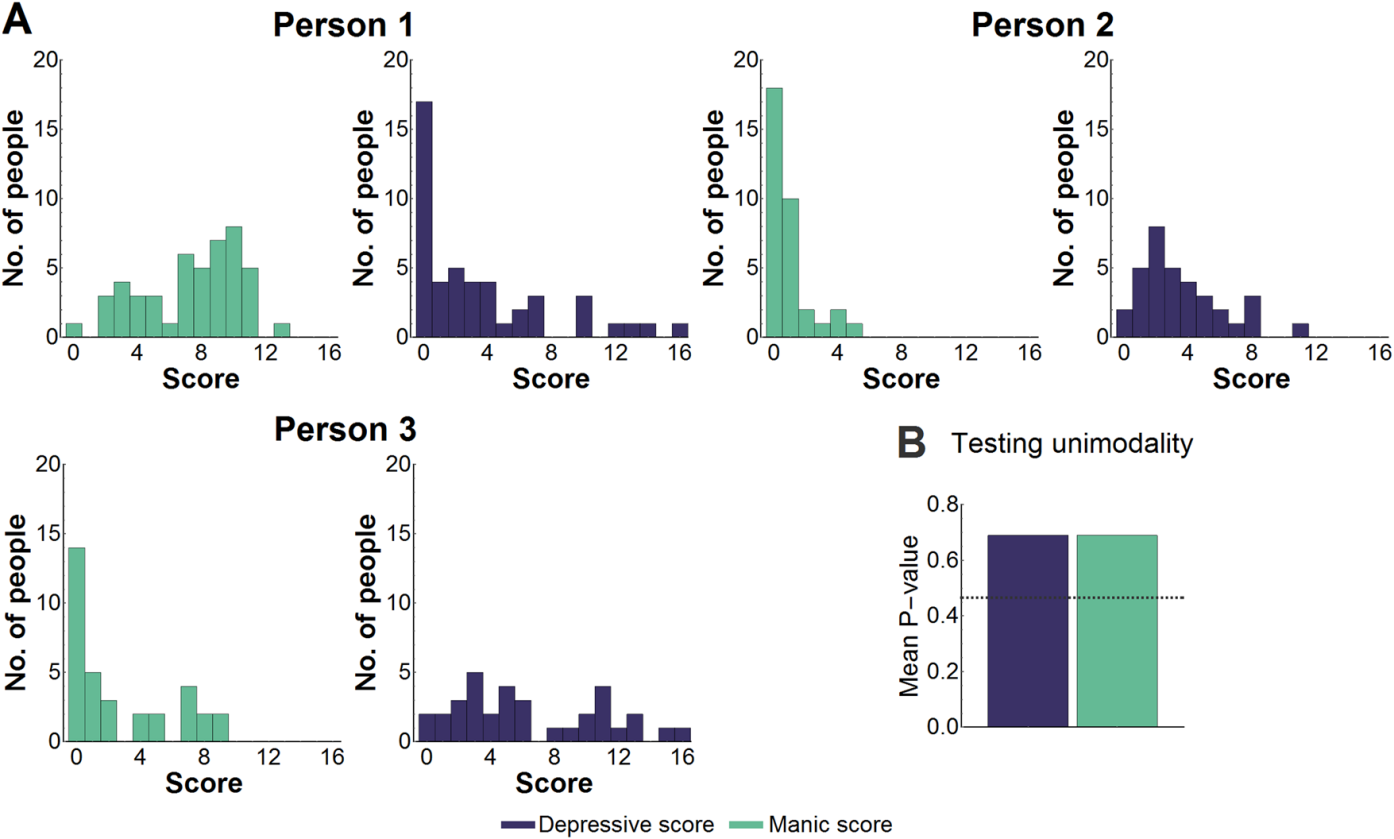
Testing a multistable hypothesis. **a** Histograms were generated from bimonthly samples of depressive and manic symptoms. A multistable assumption leads to multimodal probability density functions (pdfs). **b** The pdfs for manic and depressive symptoms did not deviate significantly from unimodality, where significance is a mean *P*-value that dips below the solid line, which mark 5th quantiles for the mean of *N* uniform random variables between 0 and 1 with *N* = 178 for the bimonthly dataset

Under a multistable assumption, an individual has multiple mood values that are relatively more stable than nearby mood values and hence would spend relatively more time around these values than nearby values. This feature would manifest as multiple modes in its probability distribution, where each mode would represent a mood value relatively more stable than nearby moods (Fig. 3a). So, if we determined that the data allowed us to reject a unimodal distribution, then we could conclude that a multistable model was appropriate. Combining tests across patients, we could not reject unimodal distribution for bimonthly manic or depressive symptoms ($\hat P_{ii}$ > 0.50; Fig. 3b).

### An alternative hypothesis for mood in BP

**Fig. 4:**
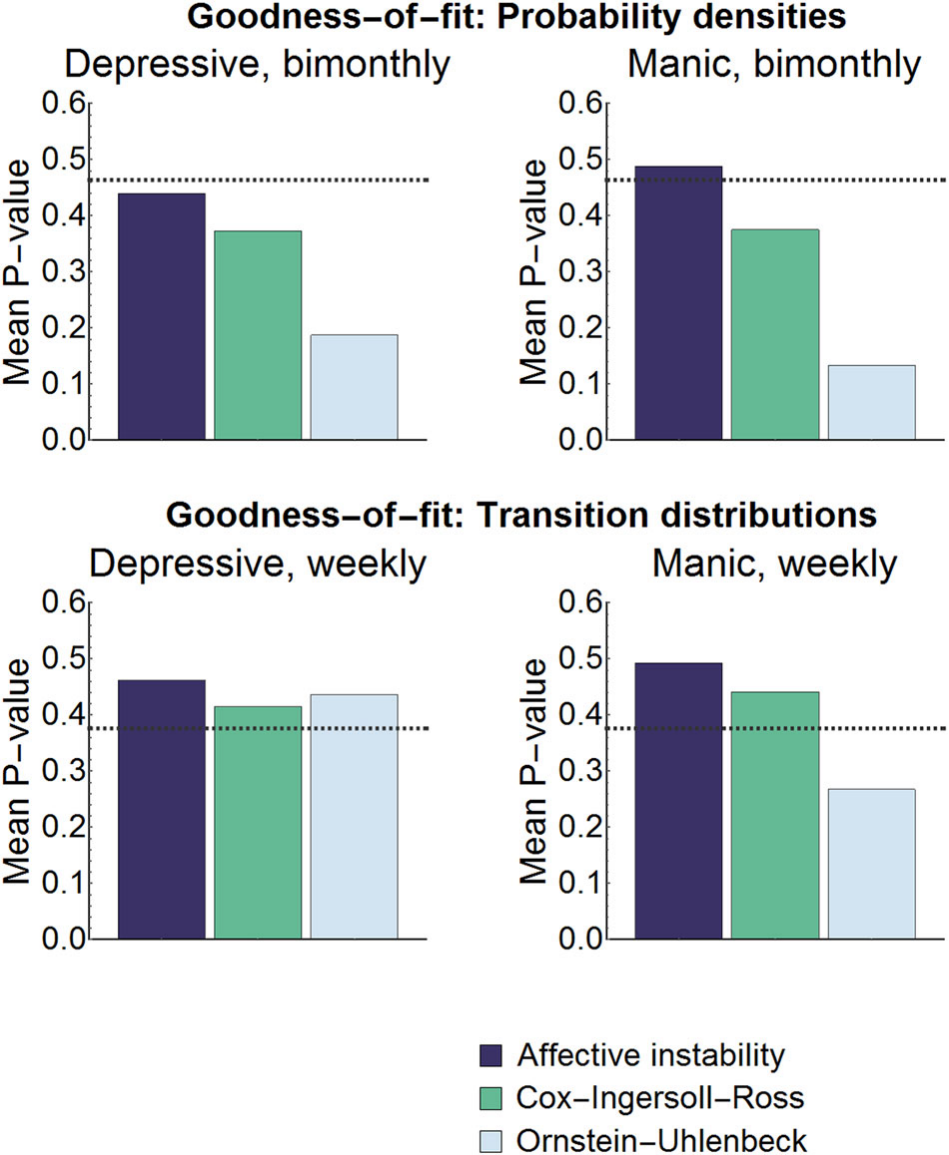
Testing the affective instability model. Goodness-of-fit tests were used to see if certain models could explain actual mood data as well as simulated data from the model. Actual and simulated data were fit to density functions and transition distribution functions associated with the affective instability model and two other popular models, an Orstein-Uhlenbeck and Cox-Ingersoll-Ross model. Except for density functions of bimonthly depressive symptoms, we found no significant differences between actual mood data and data simulated from the affective instability model. Significance is a mean *P*-value that dips below the dashed lines, which mark 5th quantiles for the mean of *N* uniform random variables between 0 and 1 with *N* = 178 for the bimonthly dataset and *N* = 15 for the weekly dataset

Not being able to reject unimodality, we questioned if a model with only one stable point (in the absence of noise) could explain the mood data. With goodness-of-fit tests, we determined if we could distinguish between actual mood data and data sampled from three *unistable* models: our affective instability described below and two popular models from finance, an Orstein-Unhlenbeck model and Cox-Ingersoll-Ross model. On average, bimonthly manic symptoms fit the density function associated with our affective instability model as well as 49% of sampled data, and bimonthly depressive symptoms fit the density function associated with our model as well as 44% of sampled data (Fig. 4). As a result, we did not find a significant difference between bimonthly manic symptoms and data simulated from our model ($\hat P_{iii}$ = 0.29). Even though the model did as well as 44% of sampled data, we could find a significance difference between bimonthly depressive scores and data sampled from our model ($\hat P_{iii}$ = 0.003). However, agreement between model and data was much higher for the affective instability model than for density functions associated with the other two models, where we could find significant differences in all cases ($\hat P_{iii}$ < 1e-9).

We also tested if sequences of weekly manic and depressive symptoms could fit *transition* distributions (i.e. the distribution function for a mood value at some point in time conditional on a preceding value) associated with each model as well as simulated data. On average, weekly manic symptoms fit the transition distribution associated with our model as well as 49% of sampled data, and weekly depressive symptoms fit the density function associated with our model as well as 46% of sampled data (Fig. 4). Hence, we did not find a significant difference between sequences of weekly manic symptoms and sequences sampled from our model ($\hat P_{iii}$ = 0.46) or between sequences of weekly depressive scores and sequences sampled from our model ($\hat P_{iii}$ = 0.31). Agreement between model and data was also found for the other two models in the case of sequences of weekly depressive scores ($\hat P_{iii} > 0.12$) and for the Cox-Ingersoll-Ross model in the case of sequences of weekly manic scores $\hat P_{iii} = 0.22$, but not for the Ornstein-Uhlenbeck model in the case of sequences of manic depressive scores ($\hat P_{iii} =$7e-4). Overall, the affective instability model best explained the data.

Note that our affective instability model has only one stable state, and so, its density function and transition distributions are both unimodal. For a model with multiple stable states, an individual’s mood *conditional on a particular starting value* would spend more time around certain mood values relative to nearby scores (similar to its density function), which manifests as multiple modes in its transition distributions. Not being able to reject specific unimodal density functions and unimodal transition distributions for our datasets provides further evidence against a multistable assumption.

### An affective instability model

Motivated by the empirical results, we describe mood as a two-dimensional random process represented by a depressive variable *D*_*t*_ and manic variable *M*_*t*_. These variables take positive values ranging from zero for no symptoms, to a larger number for minor symptoms, to an even higher number for severe symptoms; and satisfy the stochastic differential equation (SDE) model:

$$dD_t = a_d\left( {\frac{{b_d}}{{D_t}} - D_t} \right)dt + \sqrt {2a_d} \left( {\sqrt {1 - \rho ^2} dV_t + {\mathrm{\rho }}dW_t} \right)$$

$$dM_t = a_m\left( {\frac{{b_m}}{{M_t}} - M_t} \right)dt + \sqrt {2a_d} dW_t$$

where $a_d,a_m,b_d,b_m$ are positive parameters, $\rho \in [ - 1,1]$, and *dV*_*t*_ and *dW*_*t*_ are independent Wiener processes. A Wiener process, also known as Brownian motion, is the most standard way to mathematically model a continuous timecourse that is noisy. Lastly, mood variables are related to particular survey scores through scaling: *s*_*d*_*D*_*t*_ and *s*_*m*_*M*_*t*_, for positive parameters *s*_*m*_ and *s*_*d*_.

**Fig. 5:**
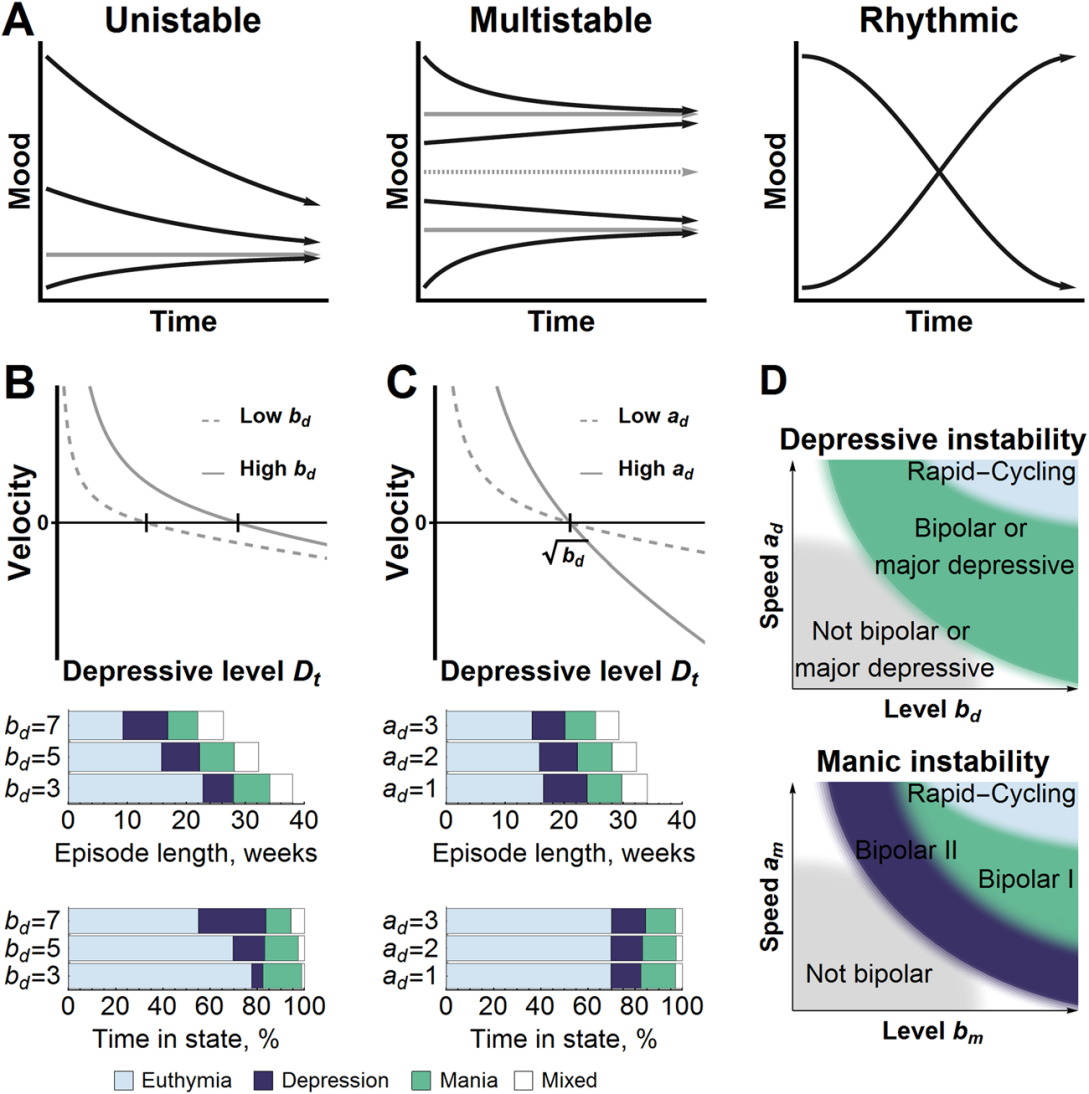
Understanding the affective instability model. **a** Ignoring noise and external perturbations, mood in the affective instability model tends to one stable state, regardless of its starting value. In a multistable model, mood tends to stay high or low, depending on its starting value, and in a rhythmic model, mood tends to oscillate between high or low values. **b**, **c** The affective instability model can be customized to an individual with longer depressive episodes and shorter non-episodic states by increasing *b*_*d*_ (dimensionless) and shorter depressive and non-episodic states by increasing *a*_*d*_ (in units 1 per 1000 days). Changes to *b*_*d*_, *a*_*m*_ have an analogous effect on manic symptoms. Unless otherwise specified, *a*_*d*_ = 2 per 1000 days, *b*_*d*_ = 5, *a*_*m*_ = 3 per 1000 days, *b*_*m*_ = 5, *ρ* = 0. **d** A caricature of how the affective instability model does not impose rigid boundaries between diagnoses. Continuous changes to *b*_*m*_*a*_*m*_*b*_*d*_ and/or *a*_*d*_ can recover a mood course that is more characteristic of an individual with either bipolar I, bipolar II, rapid-cycling, major depressive disorder, or none of these disorders

From the model, we can see that mood is inclined towards a baseline/normal level, represented by a single asymptotically stable point $(\sqrt {b_d} ,\sqrt {b_m} )$ in the absence of noise (Fig. 5a), but reaches pathological levels in certain individuals for one of two reasons. Either the baseline level is simply too close to pathological levels, so that even small fluctuations can bring mood into pathological levels. Or, mood is particularly sensitive and reactive to (random/unobserved) events, e.g. stressful life events such as job loss. Simply put, clinical course in BP arises from a weaker ability to keep mood within normal ranges. Note that the model does not impose any boundaries between mood states, such as in a multistable model (Fig. 5a). Without a natural boundary, sub-threshold symptoms, i.e. those insufficient in number or criteria to constitute a mood episode, have increased importance. As in actual individuals with BP, sub-threshold symptoms in the model persist even when DSM mood episodes have long subsided, and persons can spend upwards of half their time with sub-threshold symptoms^27^.

The model can be personalized to an individual through parameter choices. Parameters *b*_*d*_ and *b*_*m*_ influence the overall severity of mood (Fig. 5b). Higher values of *b*_*d*_ captures an individual that spends more time with depressive symptoms and has longer depressive episodes. Higher values of *b*_*m*_ has an analogous effect on manic symptoms. Parameters *a*_*d*_ and *a*_*m*_ control the speed at which symptoms fluctuate (Fig. 5c), where higher values of *a*_*d*_ or *a*_*m*_ lead to shorter mood episodes to reflect a rapid-cycler. The parameter *a*_*d*_ is dimensional compared to the current definition of rapid-cycling, which as a categorical variable may impose an artificial boundary between patients^28–30^. Parameter *ρ* controls the prevalence of mixed states, ranging from *ρ* = −1 to 1 for manic symptoms that oppose or cooperate with depressive symptoms, respectively. *ρ *= −1 would indicate that manic and depressive symptoms are not correlated. Lastly, *s*_*d*_ and *s*_*m*_ determine how mood translates into external observations, capturing, for example, variation in how mood is measured between surveys, clinicians, and/or demographic groups (gender, culture).

Although we focus on BP, there is no assumption in the model that excludes it from describing anyone’s mood. By adjusting parameters, we can theoretically capture mood courses that describe not only bipolar I, bipolar II and rapid-cycling, but also disorders such as major depression (low *b*_*m*_ and high *b*_*d*_) as well as healthy individuals (Fig. 5d). Major mood disorders can thus be conceptualized with boundaries that are more fluid than those recognized in the DSM.

## Discussion

Mathematical modeling provides an important framework for understanding human behavior. Borbély’s two-process model has formed the basis for how many researchers reason about sleep-wake dynamics^31^, and Daan’s evening-morning oscillator model likewise has led to an important understanding of how behavior can be consolidated in a 24-h day^32^. Both papers remain high cited, and many similar high-level models are used to understand physiological processes. Based on the success of these previous models, a framework for mood dynamics in BP could be impactful for the field.

A major challenge in the study of BP is how to properly classify patients, which has led to much debate about the DSM-5. A recent study has shown that bipolar I patients can be fit to a mathematical model^33^, and that the parameters of these fits can be classified into three groups with validated clinical outcomes. Our model has only seven parameters, which can easily be identified from patient data. As it is a framework that is validated against data, it should provide an important diagnostic tool for bipolar subjects.

We examined hypotheses about mood course in a dataset of manic and depressive surveys from 178 BP subjects followed for a minimum of four years. Patient-level statistics were combined to formally test these hypotheses at the patient-level. This analysis finds little evidence in our data of one-dimensionality, rhythmicity, or multistability, but finds support for an affectivity instability model. Data with higher temporal resolution, longer sampling periods, and additional patients could provide additional testing of modeling hypotheses. For example, one could test the possibility that mood should be modeled differently during different pharmacological treatments, or as the disease progresses. The bimonthly dataset was self-reported, and hence, subject to reporting bias. The weekly dataset, for example, was based on trained interviewer assessments and reinforced conclusions from the bimonthly dataset. Our model also assumes mood is Markovian, meaning that the current mood state is all that is needed to predict future mood and parameters do not vary with time. Future research might also incorporate events, such as a job loss, into the model as was done by Steinacher and Wright^11^.

Three additional modeling frameworks have also been proposed, but for which further testing does not appear to be needed. Mood was proposed to be generated by a mathematical chaotic system^34^, which is difficult to distinguish from randomness with discrete samples^35^. Nevertheless, previous testing of this hypothesis has questioned chaos’ role in mood disorders^36–38^. Other models are built just the rate of transitions between mood states rather than mood on a dimensional scale. Although they do not fulfill the modeling goals of this manuscript, such models are useful for classifying patients^33^. “Kindling” models of Bipolar have also been proposed, but the kindling hypothesis also has come into question^39^.

The affective instability model provides an alternative hypothesis for how biological processes could drive mood in BP, namely, manic and depressive symptoms could be driven by a two-dimensional process that is weakly regulated. Mania and depression are separately regulated, but may respond to some similar unpredictable or random inputs or environmental factors. Other random models of mood in BP have been presented where time and mood can be discrete or continuous^4,11,13,20,33,36,40–42^. A continuous-time continuous-state model, such as the one introduced here and those presented in Bonsall et al.^4^ and Steinacher and Wright^11^ permits data at regular or irregular time intervals and from different surveys. Random models, as the foundation of statistical inference, are relatively easy to personalize and validate with data, whereas inference is less common for chaotic models.

A major benefit of the affective instability model is that it provides a quantitative and dimensional phenotype for studying BP. Not only is mood characterized on both a manic and depressive scale, but each of the model parameters could be viewed as a characterization of an individual’s illness. Dimensional constructs that capture both pathological and non-pathological behavior are emphasized in RDoC from the National Institute of Mental Health^43^, since current classification categories are believed to impose artificial boundaries between individuals. Future research could use the affective instability model by customizing model parameters to any individual’s behavior and then utilizing these parameters to explain variation in behavior between subjects. The resulting seven parameters also provide a meaningful way for a clinician to assess clinical courses, e.g. assess the tendency for mixed episodes. With roles in BP’s pathophysiology^1^, potential candidates for the biological processes that could lead to an affective instability model would be the serotonergic and dopaminergic systems providing an important potential link to physiology.

## Electronic supplementary material

Supplementary Appendix
