# Supplementary material for: Testing frameworks for personalizing bipolar disorder
Source: Transl Psychiatry. 2018 Feb 2;8:36. doi: 10.1038/s41398-017-0084-4 (PMC5804032; doi:10.1038/s41398-017-0084-4)
Supplement: Supplementary file 1 — Supplementary Appendix [file 41398_2017_84_MOESM1_ESM.docx]

Appendix

## *Nonparametric goodness-of-fit tests*

In what follows, we provide further details on non-parametric tests used to examine two types of null hypotheses:

- Null hypothesis iii: *An individual’s manic or depressive scores has a specified unimodal density function.*
- Null hypothesis iv: *An individual’s manic or depressive scores has a specified unimodal transition distribution.*

For null hypothesis iii, we measured goodness-of-fit between the bimonthly dataset and stationary probability density functions (pdfs) from the affective instability model (defined in the main text), an Ornstein-Uhlenbeck model or a Cox-Ingersoll-Ross model ([1](#_ENREF_5)). For null hypothesis iv, we measured goodness-of-fit between the weekly dataset and the transition distribution from each of the same three models. In all cases, we assume manic and depressive scores are independent. For the Cox-Ingersoll-Ross and affective instability models, we also shifted the data up by 1/3 to avoid numerical instability that arises due to zero-valued survey scores.

For each null hypothesis, distribution, and individual, a P-value was calculated for a test statistic following an approach described in ([2](#_ENREF_6), [3](#_ENREF_7)). The approach consists of six steps:

1. Approximate the distribution/density function *parametrically* from N data points,
2. Approximate the distribution/density function *non-parametrically* from the same data,
3. Measure error between the non-parametric and parametric approximations,
4. Generate 1000 samples of N data points from the parametric approximation,
5. For each sample, repeat steps 1-3 replacing original data with sampled data,
6. Calculate P-value, i.e. probability that error from the data is less than error from samples.

For the first step, unknown parameters were estimated from data using maximum likelihood estimation (MLE). For the stationary pdfs, we used Matlab’s *mle* function to estimate parameters of the pdfs. The affective instability model has stationary pdf that is a special case of a generalized gamma pdf, a Cox-Ingersoll-Ross model has a Gamma stationary pdf, and an Orstein-Uhlenbeck model has a normal stationary pdf. For the transition distributions, parameters were estimated for the Cox-Ingersoll-Ross model based on the approach in ([4](#_ENREF_8)). We applied the same approach for the affective instability model on survey scores squared, since scaling mood variables by $s_{d},s_{m}$ in the affective instability model and then squaring the scaled variables yields a Cox-Ingersoll-Ross model (which can be shown using Ito’s lemma). The same approach was then used for the Ornstein-Uhlenbeck model after modifying the likelihood function.

For the second step, densities functions were approximated non-parametrically using a kernel-based approach available with Matlab’s *ksdensity* function which expresses the functions as a mixture of normal density functions. Except for the Ornstein-Uhlenbeck model, we used the option in the Matlab function that forces the non-parametric approximation to have positive support. This is achieved by log-transforming the data, approximating the density of the transformed data with normal distributions, and then transforming the approximation back. For transition distribution functions, suppose that $\left\{ \left( x_{i},y_{i} \right):i=1,..,N \right\}$ is a collection of patient data such that $\left( x_{i},y_{i} \right)$ denotes a transition from variable $x_{i}$ at time zero to $y_{i}$ at time $t$ for fixed $t$.

A transition distribution function $P(y|x)$ was estimated non-parametrically using the formula

$$P\left( y | x \right)=\frac{\int_{-\infty}^{y} f\left( x,y \right)dy}{f(x)}\approx\frac{\sum_{i=1}^{N} f_{i}\left( x \right)F_{i}(y)}{\sum_{i=1}^{N} f_{i}(x)}$$

where $f(x,y)$ denotes the joint density function, $f(x)$ denotes the marginal density function, $f_{i}(x)$ is a kernel density function estimated using Matlab’s *ksdensity* using only $x_{i}$, and $F_{i}(y)$ is a kernel distribution function estimated using Matlab’s *ksdensity* using only the data $y_{i}$. The option to restrict functions to positive support was used for the affective instability and Cox-Ingersoll-Ross models. Fixed bandwidths were used for all the functions $f_{i}$ $i=1,\ldots,N$ and determined using Matlab’s *ksdensity* applied to the patient data $\left\{ x_{i}: i=1,\ldots, N \right\}$. Similarly, fixed bandwidths were used for all the functions $F_{i}$ $i=1,\ldots,N$ with the bandwidth determined using Matlab’s *ksdensity* applied to the data $\left\{ y_{i}: i=1,\ldots, N \right\}$.

For the third step, we estimated the error between non-parametric and parametric approximations using a weighted sum of square error. For patient data $\left\{ x_{i}: i=1,\ldots, N \right\}$ with sample mean $\mu$ and standard deviation $\sigma$, the error between a non-parametric density approximation $f(x)$ and parametric density approximation $g(x)$ was measured as

$$\sum_{i=1}^{N} w_{i}\left( f\left( x_{i} \right)-g(x_{i}) \right)^{2}$$

where $w_{i}$ was one if $\left| x_{i}-\mu\right|<2\sigma$ and zero otherwise. Similarly, for patient transition data $\left\{ \left( x_{i},y_{i} \right): i=1,\ldots, N \right\}$ with non-parametric transition distribution approximation $F(y|x)$ and parametric transition distribution approximation $G(y|x)$, the error was measured as

$$\sum_{i=1}^{N} u_{i}\left( F\left( {y_{i}|x}_{i} \right)-G(y_{i}|x_{i}) \right)^{2}$$

where $u_{i}$ was one if $\left| x_{i}-\nu\right|<2\tau$ and zero otherwise and where in this case, $\nu$ is the sample mean of the $x_{i}$ ($i=1,\ldots,N)$ and $\tau$ is the sample standard deviation of the $x_{i}$ ($i=1,\ldots,N$).

The fourth and fifth step require no extra details. For the sixth and final step, we collected the error measurements from the samples and the error measurement from the original data. We then used Matlab’s *ksdensity* function, with the option for positive support and the cumulative density function, to estimate the probability that the error for the original data was less than the error for the sampled data. This estimated probability was used as the patient-specific P-value, which we then aggregated across patients.

**References**

1. Iacus SM. Simulation and inference for stochastic differential equations : with r examples. New York, N. Y.: Springer; 2008. xviii, 284 p. p.

2. Fan JQ. A selective overview of nonparametric methods in financial econometrics. Stat Sci. 2005;20(4):317-37.

3. Ait-Sahalia Y, Fan JQ, Peng H. Nonparametric Transition-Based Tests for Jump Diffusions. J Am Stat Assoc. 2009;104(487):1102-16.

4. Kladıvko K. Maximum likelihood estimation of the Cox-Ingersoll-Ross process: the Matlab implementation. Technical Computing Prague. 2007.
